# Perinatal mortality audit in a rural referral hospital in Tanzania to inform future interventions: A descriptive study

**DOI:** 10.1371/journal.pone.0264904

**Published:** 2022-03-11

**Authors:** Paschal Mdoe, Sifaeli Katengu, Godfrey Guga, Vickfarajaeli Daudi, Ibrahim Ernest Kiligo, Joshua Gidabayda, Catherine Massay, Felix Mkini, Estomih Mduma

**Affiliations:** 1 Department of Obstetrics and Gynecology, Haydom Lutheran Hospital, Haydom, Tanzania; 2 Research, Haydom Lutheran Hospital, Haydom, Tanzania; 3 Department of Pediatrics, Haydom Lutheran Hospital, Haydom, Tanzania; 4 Research and Advocacy, Haydom Lutheran Hospital, Haydom, Tanzania; African Population and Health Research Center, KENYA

## Abstract

**Background:**

Clear evidence exists that perinatal audit and feedback can lead to important improvements in practice. The death audit can lead to the identification of existing potential delays which are the decision to seek medical care, reaching an appropriate facility, and receiving timely adequate care at the facility. Such an audit potentially initiates a positive discussion, which may foster the implementation of changes that aims at saving more lives.

**Objective:**

To review the perinatal deaths case notes and identify potential gaps in care provision and health-seeking behavior to foster best practice.

**Methods:**

The stillbirths and neonatal death case notes that occurred between January 2019 and May 2020 at the hospital were reviewed using an adapted WHO checklist. The completed review case notes were entered into an electronic system and a quality control check-up was done. Data were analysed descriptively, and findings were presented in tables.

**Results:**

There were 4,898 births, and 1,175 neonatal admissions, out of these there were 135 recorded stillbirths (2.8%) and 201(4.1% of the total hospital births) early neonatal deaths. Out of the 1,175 neonates, 635 (54%) were born within the hospital and 540 (46%) were admitted from other facilities and home deliveries. In total 86 stillbirths and 140 early neonatal deaths case notes were retrieved and audited. Out of 86 stillbirths’ case notes audited, 30 (34.9%) seemed to have died during labor, and of these 5 had audible fetal heart rate during admission. Apgar scores less than 7 at 5 minutes, prematurity, and meconium aspiration were the top three recorded causes of neonatal deaths. Inadequate/late antenatal care visits and home delivery were the maternal factors likely to have contributed to perinatal deaths. Inadequate labor monitoring (12%) and documentation (62%) were among the providers’ factors likely to have contributed to perinatal deaths.

**Conclusion:**

This audit shows that there are high rates of preventable intrapartum stillbirths and early neonatal deaths. Both women and providers’ factors were found to have contributed to the stillbirths and neonatal deaths. There is a need to encourage women to adequately attend antenatal care, utilize health facilities during birth, and improve maternity and neonatal care at the health facilities.

## Introduction

During the year 2018, about 2.5 million neonatal deaths and over 2 million stillbirths were reported among the 6.2 million deaths of children under 15 years globally [1]; 98% of these deaths occurred in low- and middle-income countries (LMICs) [2] of which a vast majority of the deaths were preventable [3]. In the regions with the highest mortality, perinatal deaths are poorly recorded and are therefore most likely to be under-reported [3]. Understanding the numbers and collecting information about where, and the underlying contributing causes especially those which are avoidable is crucial in the effort to prevent perinatal mortality [4].

There is evidence that perinatal audits and feedback can lead to important improvements in practice [5–7]. The practice of collecting accurate data on how many maternal deaths occur, where the women die, why they die, and what could be done differently to prevent similar deaths have been established in many countries globally [8], including Tanzania. Consequently, applying similar data collection processes as well as a systematic analysis of events and contributing factors leading to individual perinatal death is considered achievable and will help identify health system breakdowns and inspire local solutions to prevent such deaths in the future [9].

Causes of perinatal deaths usually are obstetric in origin and are strongly associated with the causes of maternal mortality and morbidity [10, 11]. As a result, factors associated with perinatal deaths may be an indirect measurement of the availability and quality of care provided to a woman during childbirth and to the neonate during the neonatal period [12, 13]

Lack or inadequate care during the antenatal period, prematurity, low birth weight, and primigravity have strongly been associated with perinatal mortality [14]. Obstetric complications including obstructed/prolonged labor, preeclampsia/eclampsia, and antepartum hemorrhage are among the identified causes associated with perinatal mortality [15]. The perinatal audit conducted by Kidanto et al. (2009) at Muhimbili National hospital found a high (about 80%) prevalence of suboptimal care majority of them thought to be the likely cause of the adverse perinatal outcomes [16]. The maternal and fetal monitoring practice during labor was the main suboptimal factor found in the study, also delay in referral and operative interventions were prominent [16].

There have been several efforts to improve maternity and newborn care in this setting, however, the stillbirths and neonatal death rates are still unacceptably high in the setting and the country. The three delay model, stipulating delays which are decision to seek care, reaching the health facility and receiving appropriate care, have been studied extensively. Despite the available knowledge, periodic systematic review and clinical feedback are the key to the improvement of local quality of care and inform others on the available robust solutions. We decided to conduct the perinatal audit in the rural referral hospital aiming at identifying potential gaps in care provision and health-seeking behavior to foster best practice.

## Method

### Study setting

The audit was conducted at Haydom Lutheran Hospital (HLH). HLH is in the Northcentral of Tanzania at 1700M altitude in the Manyara region, with an average of 3500 births per annum [17]. HLH serves as a regional referral hospital serving around two million people in a wide catchment area. The hospital has a neonatal ward (unit) that cares for the neonates who are born in the hospital and those referred from other health facilities. Additional to care provision, HLH is also a teaching hospital for nurse midwives, clinical officers, and laboratory technologists. HLH is also a training hospital for international elective students, internship for both nurses, medical doctors, and pharmacists. The hospital conducts regular on-job refresher training on helping babies breathe (HBB), essential newborn care, and emergency obstetrics for nurses and midwives working in the labor ward and neonatal care unit.

The auditing processes are already being used in this hospital in the form of maternal and perinatal death surveillance and response [18]. However, less information is being captured for stillbirths and neonatal deaths and includes basic information about each stillbirth and neonatal death which are not adequately captured.

### Study design

This is a cross-sectional observational study design whereby perinatal deaths case notes that occurred at HLH from January 2019 through May 2020 were retrieved and audited.

#### Study population and sample size

All births that occurred at the hospital and all neonates who were admitted at the hospital during the study period.

### Definition of terms

Stillbirths are defined according to the WHO; as the deaths of a fetus from 28 completed weeks of pregnancy in LMIC settings [2, 19]. Macerated stillbirths (antepartum stillbirths) are the deaths that occurred intrauterine sometimes before the onset of labor/delivery and the fetus show degenerative changes. Fresh stillbirths (intrapartum stillbirths) are the intrauterine death of a child that has occurred during labor or delivery. Neonatal death is the death of a live-born baby within the first 28 days of life. Perinatal mortality is the fetal death from 28 completed weeks of pregnancy and deaths of live-born neonates up to 7 completed days of life [20].

#### Data collection

Data were collected using a designated case review form (CRF) from the patient’s case notes. Information retrieved included; demographic information, antenatal care history, previous and current obstetric history; delivery care, birth, and the neonate’s treatment history. Research assistants were responsible to identify and complete the CRFs. The CRF was then reviewed by a clinician who commented on the possible cause of death and a likely contributing factor(s) based on the reviewed case notes. The research team included the obstetrician, pediatrician, and senior midwife who reviewed the comments made by a clinician. The agreed completed CRF was entered into the database for analysis. Before data collection, the research assistants were trained on how to extract the required information from the patient’s files to complete the CRF. Piloting was done to test the CRF and the ability of researchers to use CRF. Research assistants collected a standardized set of information from the patient file/records. The completed electronic CRF for each case was uploaded into the database.

### Statistical analysis

Data were analysed descriptively using numbers, percentages, means, and standard deviations to summarise the data numerically and present it in tables.

### Ethical considerations

Ethical approval was granted by the National Institute for Medical Research (NIMR) in Tanzania. HLH administration also approved the study to be conducted at HLH. The study followed a specific data protection management plan. The ethical review authority approved the retrieval of medical records; all patient records were anonymized before data extraction. All the information was stored securely at the hospital research center. A password-protected database on a hospital research computer was used as per the country’s data governing laws.

## Results

There were 4,898 births and 1,175 neonatal admissions. Out of these, there were 135 stillbirths (2.8%) of which fresh stillbirths were 60 (44.4%), and 201 neonatal deaths (4.1% of the total hospital birth and 17.1% of the total neonatal ward admission). Out of all stillbirths and neonatal deaths, 86 stillbirths case notes (63.7% of all stillbirths) and 140 neonatal deaths (death occurring within one week of life) case notes (69.7% of all neonatal deaths) were retrieved and audited. From the case audit, the mean maternal age was 27 years, and almost half (48.4%) of mothers had completed primary education. About three-quarters (73.5%) were married or living with a partner, and more than 80% were housewives (Table 1).

**Table 1:** Socio-demographic characteristics of women who encountered perinatal deaths.

	Total	Neonatal death	Stillbirths
	(N = 226)	(N = 140)	(N = 86)
**Age**			
Mean (SD)	27.2 ± 7.5	25.5 ± 6.5	28.8 ± 8.1
**Mother education level**			
Never been to school	20 (8.9%)	12 (8.6%)	8 (9.3%)
Primary	109 (48.4%)	56 (40.3%)	53 (61.6%)
Secondary	25 (11.1%)	20 (14.4%)	5 (5.8%)
Higher	0 (0.0%)	0 (0.0%)	0 (0.0%)
Vocational training	1 (0.4%)	0 (0.0%)	1 (1.2%)
others	1 (0.4%)	1 (0.7%)	0 (0.0%)
Unknown	69 (30.7%)	50 (36.0%)	19 (22.1%)
**Civil status**			
Married/Living with partner	166 (73.5%)	99 (70.7%)	67 (77.9%)
Single	15 (6.6%)	10 (7.1%)	5 (5.8%)
Divorced	0 (0.0%)	0 (0.0%)	0 (0.0%)
windowed	0 (0.0%)	0 (0.0%)	0 (0.0%)
Unknown	45 (19.9%)	31 (22.1%)	14 (16.3%)
**Mother profession**			
Formally employed	1 (0.4%)	1 (0.7%)	0 (0.0%)
Informally employed	1 (0.4%)	0 (0.0%)	1 (1.2%)
Self employed	3 (1.3%)	3 (2.1%)	0 (0.0%)
Student	1 (0.4%)	1 (0.7%)	0 (0.0%)
Unemployed/Housewife	184 (81.4%)	99 (70.7%)	85 (98.8%)
Unknown	36 (15.9%)	36 (25.7%)	0 (0.0%)
**Religious affiliation**			
Christian	189 (83.6%)	122 (87.1%)	67 (77.9%)
Muslim	12 (5.3%)	6 (4.3%)	6 (7.0%)
Pagan	15 (6.6%)	7 (5.0%)	8 (9.3%)
Unknown	10 (4.4%)	5 (3.6%)	5 (5.8%)

More than half of the stillbirths (57.8%) and early neonatal deaths (53.5%) were male gender. Most mothers (77.9%) who experienced stillbirths were multipara with a history of stillbirths, augmentation of labor; lower level of hemoglobin and had severe maternal complications. More than three-quarters (80.5%) of all women whose pregnancy ended up with stillbirths or neonatal deaths had at least one ANC attendance. Almost half (48.7%) of all stillbirths or neonatal deaths were term pregnancies and 70% were delivered vaginally (Table 2).

**Table 2:** Distribution of maternal characteristics by baby conditions.

	Neonatal death	Stillbirths	Total
	(N = 140)	(N = 86)	(N = 226)
**Gender**			
Male	81 (57.8%)	46 (53.5%)	127 (56.2%)
Female	59 (42.1%)	40 (46.5%)	99 (43.8%)
**Gravida**			
Prime gravida	47 (33.6%)	18 (20.9%)	65 (28.8%)
Multipara	91 (65.0%)	67 (77.9%)	158 (69.9%)
Unknown	2 (1.4%)	1 (1.2%)	3 (1.3%)
**History of stillbirths**	1 (0.8%)	5 (6.3%)	6 (2.8%)
**History of neonatal death**	5 (3.8%)	2 (2.5%)	7 (3.3%)
**Antenatal visits**			
No visit at all	3 (2.1%)	4 (4.7%)	7 (3.1%)
Less than 4	65 (46.4%)	33 (38.4%)	98 (43.4%)
4 and above	51 (36.4%)	33 (38.4%)	84 (37.1%)
Unknown	21 (15%)	16 (18.6%)	37 (16.4%)
**Supplementation during ANC**			
Iron	86 (61.4%)	20 (23.3%)	106 (46.9%)
Folic acid	88 (62.9%)	20 (23.3%)	108 (47.8%)
Mebendazole	60 (42.9%)	7 (8.1%)	67 (29.6%)
**Type of pregnancy**			
Singleton	121 (86.4%)	84 (97.7%)	205 (90.7%)
Twins	16 (11.4%)	2 (2.3%)	18 (8.0%)
Triplets	3 (2.1%)	0 (0.0%)	3 (1.3%)
**Gestation age**			
> = 37 weeks	71 (50.7%)	39 (45.3%)	110 (48.7%)
Below 37 weeks	57 (40.7%)	35 (40.7%)	92 (40.7%)
Unknown	12 (8.6%)	12 (14%)	24 (10.6%)
**Onset labour**			
Spontaneous	130 (92.9%)	69 (80.2%)	199 (88.1%)
Induced	2 (1.4%)	15 (17.4%)	17 (7.5%)
Elective C/S	4 (2.9%)	1 (1.2%)	5 (2.2%)
Unknown	4 (2.9%)	1 (1.2%)	5 (2.2%)
**Mode of delivery/ birth**			
SVD	97 (69.3)	62 (72.1)	159 (70.4)
Breech	2 (1.4)	1 (1.2)	3 (1.3)
Other	0 (0.0)	1 (1.2)	1 (0.4)
C/S	41 (29.3)	22 (25.6)	63 (27.9)
Unknown	0 (0.0)	0 (0.0)	0 (0.0)
**C/S specification**		62 (72.1)	159 (70.4)
Elective	5 (12.2)	1 (1.2)	3 (1.3)
Emergency	36 (87.8)	1 (1.2)	1 (0.4)
**Partograph used**	42 (30.0%)	36 (41.9%)	78 (34.5%)
**Partograph partial completed**	42 (100%)	36 (100%)	78 (100%)
**Hemoglobin level**			
Normal	47 (33.6%)	39 (45.3%)	86 (38.1%)
Mild anaemia	12 (8.6%)	16 (18.6%)	28 (12.4%)
Moderate anaemia	9 (6.4%)	10 (11.6%)	19 (8.4%)
Severe anaemia	1 (0.7%)	7 (8.1%)	8 (3.5%)
Unknown	71 (50.7%)	14 (16.3%)	85 (37.6%)
**High Bp during pregnancy**	8 (12.9%)	5 (7.7%)	13 (10.2%)
**Birth weight**			
Extremely VLBWT	4 (2.9%)	1 (1.2%)	5 (2.2%)
Large for GA	7 (5.0%)	2 (2.3%)	9 (4.0%)
Low BWT	36 (25.7%)	11 (12.8%)	47 (20.8%)
Normal BWT	57 (40.7%)	54 (62.8%)	111 (49.1%)
Very LBWT	14 (10.0%)	2 (2.3%)	16 (7.1%)
Missing	22 (15.7%)	16 (18.6%)	38 (16.8%)

A total of 30 stillbirths (34.9%) were believed to die during labor, 5 of them had audible fetal heart rate at admission. About a quarter (25.6%), of all stillbirths, had intrapartum complications, of which severe pre-eclampsia accounted for more than one-third (38.5%) (Table 3).

**Table 3:** Characteristics of stillbirths.

Conditions	Total stillbirths
	(N = 86)
**Fetal heart present on admission–n (%)**	
Yes	14 (16.3%)
Unknown	21 (24.4%)
**Timing of death–n (%)**	
before onset of labor	48 (55.8%)
during labor	30 (34.9%)
Unknown	8 (9.3%)
**Type of stillbirths**	
Fresh stillbirths	30 (34.9%)
Macerated stillbirths	49 (57.0%)
Unknown	7 (8.1%)
**Cause of death (infant)**	
Congenital	6 (7.0%)
Antepartum complications	6 (7.0%)
Intrapartum complications	22 (25.6%)
Complications of prematurity	1 (1.2%)
Nuchal cord (cord around the neck)	4 (4.7%)
Anemia in pregnancy	2 (2.3%)
Infections	2 (2.3%)
Others	1 (1.3%)
Unknown/unspecified	43 (50.0%)
**Maternal complication, if had severe maternal near miss**	
Severe post-partum hemorrhage	1 (3.8%)
Severe pre-eclampsia	10 (38.5%)
Eclampsia	5 (19.2%)
Uterine Rupture	4 (15.4%)
PPH	2 (7.6%)
Severe anemia in pregnancy	3 (11.4%)
Other	1 (3.8%)
**Maternal condition**	
Maternal complications of pregnancy	11 (12.9%)
Complications of placenta, cord, and membranes	13 (15.1%)
Other complications of labor and delivery	7 (8.1%)
Maternal medical and surgical conditions	4 (4.7%)
No maternal condition	32 (37.2%)
Unknown	20 (23.3%)

From all the neonates who died within the first week of life at HLH whose notes were reviewed and included in this study, 77 (55%) were neonates born at HLH while 63 (45%) were born outside HLH i.e. referral from other health facilities, birth before arrival and home deliveries. Birth asphyxia, defined as a Low Apgar score (< 7) at 5 minutes, Prematurity, and Meconium aspiration were the top three recorded leading causes of early neonatal deaths (Table 4).

**Table 4:** Characteristics of early neonatal death.

	Early neonatal death
	(N = 140)
**Place of birth**	
HLH	77 (55.0%)
Another health facility	39 (27.9%)
Home	19 (13.6%)
On the way to HF	4 (2.9%)
Unknown	1 (0.7%)
**Reason for admission to neonatal ward**	
Low score	58 (41.4%)
Prematurity	51 (36.4%)
Meconium aspiration	43 (30.7%)
Congenital malformation	15 (10.7%)
EONS	14 (10.1%)
Others	6 (4.3%)
**Apgar score** < 7 At 1 Minute	50 (50.5%)
**Apgar score** < 7 At 5 minute	40 (40.8%)
**Any resuscitation measures taken**	
Yes	59 (42.1%)
No	41 (29.3%)
Unknown	40 (28.6%)

The factors found to have likely contributed to perinatal deaths during the audit included inadequate or late ANC booking, 60 women (26.5%), home deliveries, 20 women (8.8%), and late arrival at the hospital, 17 women (7.5%), Table 5. There were high levels of inadequate documentation (62%), followed by inadequate labor monitoring (12%) Table 5, which are providers’ factors likely to have contributed to stillbirths and early neonatal deaths.

**Table 5:** Identified contributing factor.

	N = 226
**Woman/Family contributing factors**	
Home delivery	20 (8.8%)
Inadequate (few) and Late booking of ANC visits during pregnancy	60 (26.5%)
Late arrival at the health facility for delivery	17 (7.5%)
No ANC visits during pregnancy	5 (2.2%)
Others (Not using ART/use of herbal medicine to induce labour)	2 (0.8%)
**Facility/Provider contributing factors**	
Inadequate documentations	141 (62.4%)
Inadequate monitoring during labor	26 (11.5%)
Delayed action (Intervention or consultation)	24 (10.6%)
Inappropriate action/decision taken	10 (4.4%)

## Discussion

This perinatal audit was aimed at highlighting the current situation, gaps, and opportunities to inform future interventions. This review report on the 86 stillbirths and 140 early neonatal deaths audited during the study period. The majority of women who experienced stillbirths were found to have a history of stillbirths, lower hemoglobin levels and suffered severe maternal complications like severe preeclampsia. Apgar scores less than 7 at 5 minutes, prematurity, and meconium aspiration were the top three causes of neonatal deaths. Inadequate or late antenatal care, delivery outside the health facility that included home and birth before arrival to HLH, inadequate labor monitoring were modifiable factors found to have contributed to both stillbirths and neonatal deaths.

Studies from Tanzania report stillbirths and neonatal deaths rate at 4.2% and 3.4% at referral hospitals [15, 21]. Our study reports low rates of stillbirths but high neonatal deaths (17.1%) when compared to hospitals of the same level in the country. The high neonatal death rates may be accounted for by the high number of referrals from the surrounding facilities in the region and neonates delivered at home or before arriving at the hospital. Similarly, other studies found that women delivering at home have an increased chance of losing their newborns as compared to those delivered at the health facility [22, 23]

Complications such as anemia in pregnancy, and cord around the neck are among the identified causes of most stillbirths, similarly to other studies from Tanzania and Italy [14, 24]. This study found prematurity, birth asphyxia, and meconium aspiration as the three leading causes of neonatal deaths similar to studies in other settings [25, 26]. Correspondingly with other studies [27, 28], in our study, we found that old age, multiparity, history of stillbirths, and induction of labor were high among women who experienced stillbirths. The findings may indicate the inadequacy of maternity care and the need for specialized care for women with a history of previous pregnancy loss.

This audit identified family/woman-related contributing factors namely inadequate antenatal care which was not attending ANC at all, late ANC booking, or very few visits less than recommended 4 ANC visits. Late arrival at the health facility when in labor and home delivery was also observed. Several studies have reported the highest odds for perinatal mortality for women with inadequate ANC visits [29–31], these findings from other studies concur with our study findings. In many Sub-Saharan African countries, the leading risk factors for perinatal deaths include pregnancy-related maternal complications, preterm births, birth complications, and neonatal infections [32, 33]. Most of these risk factors can be minimized or prevented through ANC interventions. Home delivery was found to have a high risk for perinatal mortality in our study similar to studies from other settings [34, 35]. The majority of home births are not attended by qualified birth attendants thus labor is not adequately managed leading to adverse maternal and perinatal outcomes.

Unfortunately, facility birth does not necessarily guarantee perinatal survival if the services are not optimal [36]. This audit highlights several facility/ providers’ factors thought to have contributed to perinatal deaths. Inadequate labor monitoring with inappropriate labor augmentation was found to have contributed to perinatal deaths. In some case notes, it was observed that there was a wide time range between decision and action which might have led to the poor outcomes. The audit encountered a high level of missing information and poor documentation. Likewise, partograph were not used in most cases and where used, mostly were incomplete. Our study findings concur with other studies reporting similar observations [15, 17, 37, 38]. Inadequate use of partogram and poor documentation has been reported to be associated with poor perinatal outcome [39].

The findings from this audit were communicated with the care providers at this hospital, several action plans were developed for implementation. We believe this will be followed up and measured in another perinatal audit. It is known that cheap and locally affordable interventions can save many lives [40]. Furthermore, commitment coupled with periodic audits and feedback have a significant contribution to the survival of newborns.

The main limitation of this audit is a high rate of missing information and failure to verify the information because the data were collected retrospectively. We did not include birth weight to ascertain maturity and only used gestational age which sometimes may be uncertain. Additionally, the audit lacks the opinions of both women who experienced perinatal loss and healthcare providers who provided care. However, the findings can be regarded as an eye-opener and can be used to facilitate a further review, set goals towards the improvement of maternity care provision in similar settings.

## Conclusions

This audit shows that there are high rates of preventable intrapartum stillbirths and early neonatal deaths. Both women and providers’ factors were found to have contributed to the stillbirths and neonatal deaths. There is a need to encourage women to adequately attend antenatal care, utilize health facilities during birth, and improve maternity and neonatal care at the health facilities.

## Abbreviations

ART: Ant retroviral treatment
HB: Haemoglobin
GA: Gestational age
IQR: interquartile range
PPH: Postpartum haemorrhage
